# PK1 from *Drosophila* obscurin is an inactive pseudokinase with scaffolding properties

**DOI:** 10.1098/rsob.220350

**Published:** 2023-04-26

**Authors:** Thomas Zacharchenko, Till Dorendorf, Nicolas Locker, Evert Van Dijk, Anja Katzemich, Kay Diederichs, Belinda Bullard, Olga Mayans

**Affiliations:** ^1^ Institute of Integrative Biology, University of Liverpool, Liverpool L69 7ZB, UK; ^2^ Department of Biology, University of Konstanz, 78457 Konstanz, Germany; ^3^ Faculty of Health and Medical Sciences, University of Surrey, Guildford, UK; ^4^ Biosynth B.V., Zuidersluisweg 2, 8243 RC Lelystad, The Netherlands; ^5^ Department of Biology, University of York, York, UK

**Keywords:** pseudokinase, X-ray crystallography, muscle sarcomere

## Abstract

Obscurins are large filamentous proteins with crucial roles in the assembly, stability and regulation of muscle. Characteristic of these proteins is a tandem of two C-terminal kinase domains, PK1 and PK2, that are separated by a long intrinsically disordered sequence. The significance of this conserved domain arrangement is unknown. Our study of PK1 from *Drosophila* obscurin shows that this is a pseudokinase with features typical of the CAM-kinase family, but which carries a minimalistic regulatory tail that no longer binds calmodulin or has mechanosensory properties typical of other sarcomeric kinases. PK1 binds ATP with high affinity, but in the absence of magnesium and lacks detectable phosphotransfer activity. It also has a highly diverged active site, strictly conserved across arthropods, that might have evolved to accommodate an unconventional binder. We find that PK1 interacts with PK2, suggesting a functional relation to the latter. These findings lead us to speculate that PK1/PK2 form a pseudokinase/kinase dual system, where PK1 might act as an allosteric regulator of PK2 and where mechanosensing properties, akin to those described for regulatory tails in titin-like kinases, might now reside on the unstructured interkinase segment. We propose that the PK1-interkinase-PK2 region constitutes an integrated functional unit in obscurin proteins.

## Graphical abstract

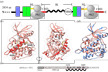

## 1. Introduction

The muscle sarcomere contains various gigantic, filamentous proteins that support the assembly and stability of its ultrastructure as well as its mechanics and homeostatic regulation [1]. Among these proteins, the obscurin family is central to sarcomere physiology [2,3]. The family comprises obscurin (Obsc), obscurin-like 1 (Obsl1) and the smaller striated muscle preferentially expressed gene (SPEG) in vertebrates. In invertebrates, a single protein exists, UNC-89/Obsc, which is a homologue of vertebrate Obsc. Obscurins are large multi-domain proteins composed of serially linked immunoglobulin (Ig) and fibronectin type-III (FnIII) domains as well as various signalling domains, including a calcium/calmodulin-binding IQ-motif, a src homology-3 domain (SH3) domain, a rho-guanine nucleotide exchange factor (RhoGEF), a pleckstrin homology (PH) domain and a C-terminal tandem of two protein kinases (PK1 and PK2) [3]. By differential-splicing, a large number of isoforms are formed with different composition of signalling domains and distinct cellular localizations [3].

Generally, obscurins localize to the periphery of the sarcomeric M-line, where they form stabilizing transversal cross-links and, further, interact with membrane structures, including the sarcoplasmic reticulum (SR) that acts as intracellular calcium storage [4–6]. The smaller obscurin homologue Obsl1 is in the core of the M-line. Notably, Obsc deficient mouse models have normal myofibril organization but disorganized SR [7]. Mammalian Obsc also localizes to Z-discs, the sarcolemma, intercalated discs and the nucleus [2,5,8] and its mutation has been linked to cardiomyopathies [9] and various cancers [10]. In *Caenorhabditis elegans*, UNC-89/Obsc localizes to sarcomeric M-bands as well as to the dense bodies of body wall muscle (the nematode's Z line analogue), which link the actin filaments to the extracellular matrix [4]. The sarcomere to SR linkage is conserved in nematode UNC-89/Obsc [11]. Mutations in the *C. elegans* gene result in disorganized sarcomeres, including a lack of M-lines, and thereby worms with compromised locomotion [11]. In *Drosophila melanogaster*, UNC-89/Obsc is present throughout the M-line but a binding to the SR is thought unlikely, since the SR is distant in the fly's indirect flight muscle (IFM), being localized half-way between M-line and Z-disc. In *Drosophila,* UNC-89/Obsc has been proven to be essential for sarcomere development [6], supporting the formation of symmetrical thick filaments and assembly of thin filaments with correct length and polarity. Downregulation of UNC-89/Obsc by siRNA resulted in IFM with abnormal M-lines and, thereby, flightless flies. Taken together, these data reveal obscurin proteins as important mediators of muscle development and function.

**Figure 1.**
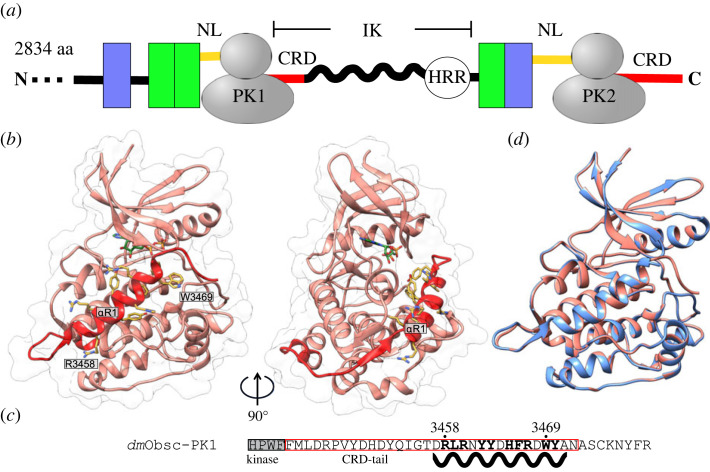
Structure of *dm*Obsc-PK1. (*a*) Domain composition of the dual kinase region of *Drosophila melanogaster* UNC-89/Obsc. Chain components: NL, predicted N-terminal linker to the domain preceding each kinase domain; CRD, predicted C-terminal regulatory domain; IK, interkinase linking segment comprising a predicted helix-rich region (HRR). Kinase domains are shown in grey; Ig domains are depicted as green boxes and FnIII domains as blue boxes. (*b*) Crystal structure of *dm*Obsc-PK1 complexed to ADP. Orthogonal views are provided. (*c*) Sequence C-terminal to the kinase domain as present in the crystallized protein sample. The motif feature corresponding to the C-terminal end of the kinase domain is shown in grey. Boxed in red is the structured CRD fraction as observed in crystal structures. The sequence spanned by helix *α*R1 is indicated by a black wavy line. Residues in bold correspond to residues explicitly displayed in the model, which primarily mediate the packing of helix *α*R1 onto the kinase domain. Tryptophan W3469 commonly linked to calmodulin-binding is annotated. (A more detailed view of helix *α*R1 and its packing residues in shown in electronic supplementary material, figure S2). (*d*) Superimposition of crystal structures of *dm*Obsc-PK1 in its apo (blue) and ADP-bound (salmon) forms (RMSD_C*α*_ = 0.18 Å for 288 aligned C_α_-atoms out of 289 total shared atoms, calculated using UCSD Chimera; https://www.cgl.ucsf.edu/chimera). No significant changes in side chain rotamers are observed between the two structures.

Understanding the role of obscurin proteins in cell signalling requires unveiling the function of the conserved dual kinase region. This region contains two kinase domains, PK1 and PK2, joined by an interkinase segment that consists of a long (typically 150–650 residues) intrinsically disordered sequence (IK) followed by Ig and FnIII domains (figure 1*a*). PK1 and PK2 belong to a distinct branch of the calcium/calmodulin(CAM)-dependent protein kinase (CAMK) family, termed the DMT class, which largely comprises cytoskeletal kinases [12–15]. In addition to obscurin kinases, the DMT class includes myosin light-chain kinases (MLCK), death associated protein kinases (DAPK) and the sarcomeric titin- and titin-like kinases. DMT kinases share the hallmark regulatory mechanism of CAM-kinases, namely autoinhibition by a C-terminal regulatory tail extension (CRD) that packs against the kinase as a pseudosubstrate. Kinase activation is commonly elicited by the binding of calcium/calmodulin to the CRD tail, although this is not a universal requirement of CAM-kinases, with calmodulin activation being conditional at times or even not required [14,16,17]. Well-characterized outliers within the DMT class are titin kinase (TK) and its nematode homologue twitchin kinase (TwcK), which are domains of large, elastic filamentous proteins of the muscle sarcomere. Crystal structures for human TK [18,19], TwcK from *C. elegans* [20,21] and TwcK from *Aplysia* [20] have shown that two tail extensions flank the kinase domain N- and C-terminally (termed NL and CRD, respectively) and that these tails are exceptionally well developed. The NL tail packs against the interlobular hinge region, and the CRD tail penetrates ATP pocket and active site. The synergistic action of both tails silences kinase activity [21]. In TK and TwcK, tail removal is not mediated by calmodulin [14]. Instead, tail unfolding appears to be induced by the pulling forces that emerge in the sarcomere during muscle function [19,21,22]. Support for the physiological relevance of this mechanism in muscle *in vivo* was brought recently by a study that monitored conformational changes in TwcK in freely moving transgenic *C. elegans* using FRET [23]. Force-induced tail unraveling is thought to activate phosphotransfer [22,24], alter the scaffolding properties of the kinases [25] and/or modulate their targetability by post-translational modifications such as ubiquitination [19]. Thus, TK and TwcK are regarded as sarcomeric mechanoreceptors. Being members of the DMT class and belonging to filamentous sarcomeric titin-like proteins, obscurin kinases could also be expected to be regulated by C-terminal, mechanosensory pseudosubstrate structures. However, the absence of conservation in CRD sequences across DMT kinases complicates a predictive analysis, so that the existence and exact extent of regulatory tails in obscurin kinases, their ability to serve as mechanosensory structures and their interaction with calmodulin remain unclear. For example, PK1 kinases of mammalian Obsc and SPEG contain a CAM-binding motif C-terminal to the kinase domain and, thereby, a predicted autoinhibitory CRD, pointing to a classical CAM mechanism of regulation [14,15]. By contrast, sequence analysis of PK2 in SPEG suggests the absence of a regulatory CRD [14] and analysis of PK2 in vertebrate Obsc as well as PK1 and PK2 from invertebrate UNC-89/Obsc could not identify CAM-binding motifs or clarify the existence of CRD extensions. These differences indicate that regulation may vary across obscurin kinases and have led to speculate on the possible loss-of-function of some members of the family, where a kinase within the tandem might remain functional while the other has become a mere evolutionary remnant [15].

The functional study of obscurin kinases is further complicated by their occurrence both as canonical kinases and pseudokinases that lack one or more of the signature motifs of ATP binding and catalytic phosphotransfer [26]. PK1 and PK2 from mammalian Obsc and SPEG are canonical kinases and their phosphotransfer activity has been experimentally demonstrated *in vitro* (reviewed in [27]). By contrast, a sequence analysis of invertebrate UNC-89/Obsc revealed that PK1 is a pseudokinase, while PK2 is probably active [26,28]. PK1 and PK2 kinases from both nematode and fly are also known to act as scaffolds. In *C. elegans*, UNC-89/Obsc kinases interact with Lim-9/FHL and the protein phosphatase Scpl-1 [29]. In *Drosophila*, PK1 binds the kinase Bällchen (Ball), and both PK1 and PK2 bind the ankyrin-repeat protein, MASK [30]. The substrate for the Ball kinase in muscle is unknown; whereas MASK has been linked to Corkscrew in a pathway leading to myogenesis and is part of the Hippo pathway, which is crucial for muscle development [31]. Downregulation of either Ball or MASK results in severely disorganized sarcomeres and flies with reduced viability [30]. In brief, UNC-89/Obsc kinases act as scaffolds that recruit factors essential for the development and maintenance of the sarcomere.

A last element of relevance in the dual kinase region of obscurins is the intrinsically disordered interkinase (IK) sequence. In mammalian Obsc and SPEG, the IK sequence is phosphorylated by up-stream kinases and in Obsc (but not SPEG), it is also autophosphorylated by PK1 [32]. Interestingly, PKB-mediated phosphorylation of the IK segment activates PK2 in SPEG, regulating its activity on the Serca2 substrate [33]. In *C. elegans*, the IK segment of UNC-89/Obsc has been found to act as scaffold, harbouring a second binding site for Lim-9 [29] as well as being shown to respond to mechanical pulling as an entropic spring, with deletion mutants lacking the IK region suffering from severe defects in sarcomere organization and locomotion [34]. Taken together, the data lead to suspect a regulatory role also for the IK sequence, but how this relates to PK1 and PK2 kinases is unknown.

Currently, little is known of the molecular mechanisms of obscurin kinases, a central question being the functional relationship of PK1 and PK2 and whether they act independently, redundantly or in synergy. To advance this knowledge, we have studied PK1 from *D. melanogaster* UNC-89/Obsc (*dm*Obsc-PK1) that has an established link to muscle development. Our data show that this pseudokinase shares overall features with members of the DMT class of CAM-kinases, but that it carries a minimalistic CRD tail that does not bind calmodulin or acts as mechanosensory element. *dm*Obsc-PK1 presents a strongly diverged active site that retains high-affinity ATP-binding properties but in a cation independent fashion. The kinase is inactive under our experimental conditions. Its highly modified peptide binding site points to the possibility that it might have evolved the ability of accommodating unconventional binders, possibly in support of its scaffold role. Finally, our data suggest that PK1 interacts with PK2, indicating that it holds a functional relation to the latter, potentially acting as its allosteric regulator. These findings lead us to speculate that PK1/PK2 form a pseudokinase/kinase dual system, where PK1 acts as regulator of PK2 and where allegedly the connecting IK sequence plays a mechanosensory role. Thus, we postulate that the PK1-IK-PK2 segment works as a functional unit in the sarcomere.

## 2. Results

### 2.1. *Dm*Obsc-PK1 lacks a developed C-terminal tail

In sarcomeric titin-like kinases, N- and C-terminal tails pack against the kinase domain and critically contribute to its stability, with the recombinant production of these kinases being highly sensitive to domain composition [35]. *dm*Obsc-PK1 is joined to the preceding Ig domain by an N-terminal linker sequence of 27 residues and is followed C-terminally by a unique sequence of 208 residues expected to contain the CRD tail plus the intrinsically unstructured IK segment (figure 1*a*). Given the difficulty of defining domain borders in this case, our study of *dm*Obsc-PK1 tested eight different protein constructions spanning the kinase domain plus a range of N- and C-terminal flanking segments (electronic supplementary material, figure S1). The recombinant production of each construction was performed in six different expression vectors, directed to explore the effect of solubility tags on expression (electronic supplementary material, figure S1). Of the variants tested, only the protein construct encompassing the kinase domain followed by 40 C-terminal residues (*dm*UNC89/Obsc residues 3186–3480) yielded a soluble and stable protein product viable for structural analysis.

The atomic structure of *dm*Obsc-PK1 was then elucidated to 1.75 Å resolution using X-ray crystallography (table 1). The structure revealed the kinase domain followed by a short CRD tail, limited to the first regulatory helix, *α*R1 (nomenclature as in 18), with the remaining 8 terminal residues being poorly ordered in electron density maps (figure 1*b,c*). Helix *α*R1 is amphipathic and its packing against the kinase is mediated by a cluster of aromatic residues that includes the conserved tryptophan W3469 at the helix C-terminus (figure 1*b*; electronic supplementary material, figure S2). A conserved tryptophan at this position is a hallmark for calmodulin binding [15]. However, this residue is commonly preceded by a cluster of positively charged residues that is part of the consensus motif for calmodulin binding in CAM-kinases (electronic supplementary material, figure S2). As illustrated by the crystal structure of DAPK in complex with calmodulin [36], a lysine residue immediately preceding the conserved tryptophan binds into a negatively charged pocket in calmodulin, being an important component of the interaction. Instead, in *dm*Obsc-PK1, an aspartate residue precedes the conserved tryptophan (figure 1*c*; electronic supplementary material, figure S2). This aspartate does not appear compatible with calmodulin binding as displayed by DAPK. In agreement with this deduction, we have not observed calmodulin binding to *dm*Obsc-PK1 in a yeast two-hybrid screening of muscle binders [30] or in tandem affinity purification from muscle extracts. Thus, we conclude that *dm*Obsc-PK1 carries a degenerated motif in its helix *α*R1 that has lost the ability to bind calmodulin.

**Table 1.** X-ray diffraction data and model refinement statistics. GOL, glycerol; TRS, 2-amino-2-hydroxymethyl-propane-1,3-diol.

	PK1 apo	PK1/ADP
PDB code	8AK2	8AK3
space group	P6_3_22	P6_3_22
cell dimensions: a,b,c (Å)	95.42, 95.42, 164.77	95.42, 95.42, 164.60
copies in ASU	1	1
data processing		
beamline	I03 (Diamond)	I03 (Diamond)
detector	PILATUS 2M	PILATUS 2M
wavelength (Å)	0.920	0.980
resolution (Å)	29.21–1.75 (1.77–1.75)^a^	29.16–1.90 (1.92–1.90)^a^
no. reflections	45 374 (1485)	35 642 (1080)
R_sym_(I) (%)	11.4 (303.0)	15.7 (337.1)
⟨I/σ(I)⟩	17.89 (0.90)	14.83 (1.14)
CC1/2	99.9 (34.2)	99.8 (31.2)
completeness (%)	99.9 (99.0)	100 (99.9)
multiplicity	13.12 (9.45)	12.53 (9.28)
model refinement		
no. working/free reflections	45 356/2253	35 621/1771
Rwork/Rfree (%)	17.38/19.59	17.35/20.78
no. atoms protein	2407	2449
no. atoms solvent	334 + 8 × GOL, 2 × TRS	215 + 5 × GOL
R.m.s.d. bond length (Å)	0.007	0.008
R.m.s.d. angles (°)	0.81	1.05
Ramachandran plot		
favoured/disallowed (%)	95.82/0	94.86/0.68

^a^Values in parentheses correspond to the highest-resolution shell.

The overall structure of *dm*Obsc-PK1 resembles that of other CAM-kinases, e.g. DAPK, which also displays a CRD tail that is disordered beyond helix *α*R1 [15]. However, this disinhibited state of *dm*Obsc-PK1 -where ATP and substrate binding sites are exposed- contrasts markedly with structures of nearest homologues, the sarcomeric titin-like kinases. In the latter, the CRD tail also contains helix *α*R2, which penetrates and blocks the ATP-binding pocket, and β-strand *β*R1 that packs against the activation loop, blocking the peptide substrate-binding site. To ensure that the lack of a fuller inhibitory CRD tail in *dm*Obsc-PK1 was not the result of premature sequence truncation in the construct, we generated 7 additional variants where the protein was extended C-terminally by 15 to 70 additional residues in progressive increments. Considering the pre-existing 40 residue long CRD sequence, this resulted in the longest of the variants spanning 110 residues C-terminal to the kinase domain; well over the length of the CRD extension of titin-like kinases (approx. 60 residues). Of all extended constructs, only that extended by 15 additional residues (*dm*Obsc-PK1^ext^) yielded a soluble recombinant protein product, which might be an indication of the alleged unstructured nature of the additional sequence. A crystal structure of the *dm*Obsc-PK1^ext^ construct could not be obtained. Hence, we explored instead the conformation of this construct by three-dimensional-modelling in AlphaFold [37]. In agreement with the crystal structure, AlphaFold models also showed a CRD tail limited to helix *α*R1 and predicted the remaining sequence to pack poorly against the kinase, being disordered and detached from the kinase domain (electronic supplementary material, figure S3). Thus, it is likely that in *dm*Obsc-PK1, helix *α*R1 with its strongly aromatic interface to PK1 (electronic supplementary material, figure S2), is retained for fold stability, but that it lacks a regulatory function as it does not occlude any of the prospective functional sites of the kinase. Supporting a role of helix *α*R1 in kinase stability, two constructs were tested in our study that ended C-terminally in residue 3444 and, thus, included the full kinase domain but lacked helix *α*R1 (electronic supplementary material, figure S1). Specifically, the construct spanning residues 3186–3444 can be regarded as a Δ-helix*α*R1 version of construct 3186–3480 corresponding to the crystal structure. Neither of the two constructs lacking the C-terminal tail resulted in the production of a soluble protein product, supporting the view that helix *α*R1 contributes to the stability of *dm*Obsc-PK1. This deduction agrees with observations on the related muscle kinases, human TK and *C. elegans* TwcK, where the removal of helix *α*R1 leads to a considerable loss in kinase stability [35]. In brief, our data led us to deduce that *dm*Obsc-PK1 lacks a bona fide, fully developed CRD extension and, thus, that this kinase does not share the intrasteric inhibition and mechanosensory mechanism of sarcomeric titin-like kinases.

### 2.2. *Dm*Obsc-PK1 adopts a catalytically primed conformation

**Figure 2.**
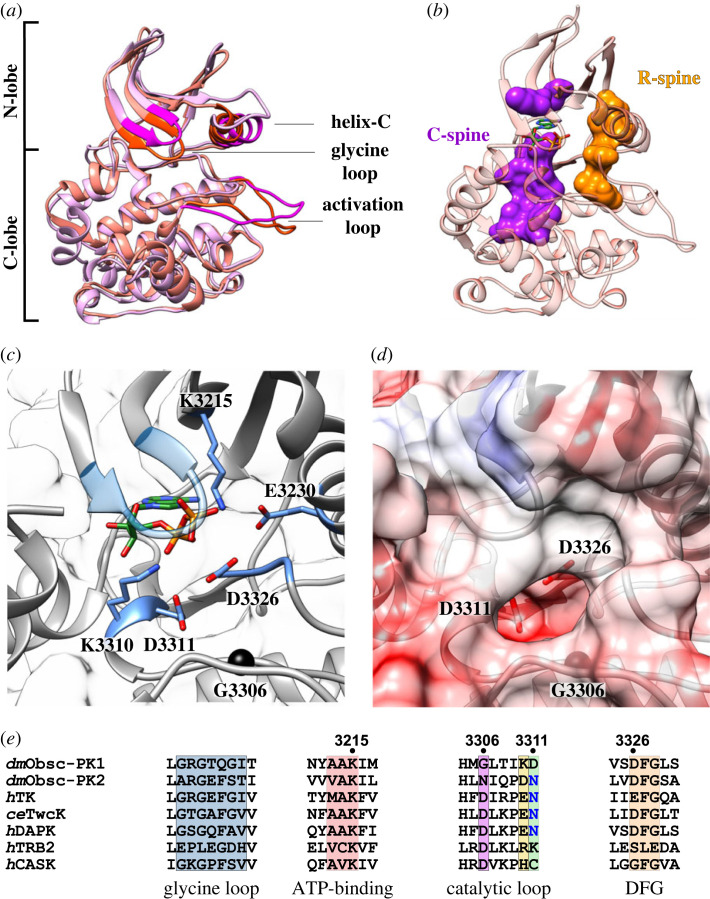
Characterization of the active site of *dm*Obsc-PK1. (*a*) Superimposition of the kinase domains of *dm*Obsc-PK1 (salmon) and human DAPK (pink; PDB entry 2W4K). (*b*) Hydrophobic spine networks in *dm*Obsc-PK1. The R-spine (residues: M3234, P3245, H3304, F3327) is shown in orange and the C-spine in purple (residues: I3199, A3213, L3267, L3312, L3313, I3314, I3371, L3375.). The adenine nitrogenated base moiety of ADP completes the C-spine. (*c*) Active site of *dm*Obsc-PK1 in the ADP bound state (ADP is shown in green). Catalytically relevant residues described in the main text are shown. Glycine 3306 is shown as a black sphere. (*d*) Surface representation of the active site of *dm*Obsc-PK1 showing the cavity resulting from the exchange of the conserved aspartate for glycine 3306. (*e*) Sequence alignment of functional motifs in representative kinases and pseudokinases from the CAMK family. *dm*, *Drosophila melanogaster*; *ce*, *Caenorhabditis elegans*; *h*, human. Kinases: TK, titin kinase; TwcK, twitchin kinase; DAPK, death-associated protein kinase; TRB2, Tribbles (TRB)-related (pseudo)kinase 2; CASK, calcium/calmodulin (CaM)-activated serine-threonine kinase. The asparagine residue in the catalytic loop that is engaged in cation coordination is highlighted in blue.

In kinases, the arrangement of N- and C-lobes, the glycine-rich loop, the catalytic helix *α*C, the activation loop and two structural hydrophobic networks termed spines adopt dynamic conformations that correlate with catalytic readiness [38,39]. ATP binding commonly induces the adoption of a catalytically active conformation characterized by an assembled regulatory (R)-spine that is often accompanied by a salt bridge formation between the conserved glutamate residue from helix *α*C (that coordinates magnesium in kinases) and the essential lysine from β-strand *β*3 (that optimally positions the non-transferable α- and β-phosphate groups of ATP) [38]. An analysis of these features in apo d*m*Obsc-PK1 referenced to DAPK2 complexed to ATP [40], revealed that the *dm*Obsc-PK1 conformation resembles that of active canonical kinases. Specifically, the N-terminal lobe of *dm*Obsc-PK1 is in a ‘closed’ conformation (figure 2*a*), where the catalytic helix *α*C is tilted into a lower position so that its conserved glutamate E3230 closely interacts with the conserved lysine K3215 in strand-β3 (2.76Å between atom NZ from K3215 and atom OE2 from E3230). In d*m*Obsc-PK1, both the catalytic (C)-spine (residues I3199, A3213, L3267, L3312, L3313, I3314, I3371, L3375) and the regulatory (R-)spine (residues M3234, P3245, H3304, F3327) are pre-formed in the absence of ATP binding. Phenylalanine F3327, part of the R-spine, belongs to the DFG invariable motif. In this ‘in’ conformation, it contributes to spine formation and, thereby, to the alignment of N- and C-terminal lobes and the correct configuration of the active site. As in canonical kinases, the d*m*Obsc-PK1 R-spine is anchored into the C-terminal kinase lobe via a conserved interaction between a histidine residue (H3298, immediately next to the R-spine member M3297) and an aspartate (D3364) in the long helix F. In this regard, d*m*Obsc-PK1 conformation agrees with that of DMT kinases characterized structurally to date, which also show constitutively assembled C- and R-spines, DFG motifs in ‘in’ conformation and a primed position of helix *α*C [15]. Finally, in d*m*Obsc-PK1*,* the activation loop is in an open state so that access to the active site is unrestricted. This agrees with the fact that DMT kinases are not known to undergo phosphorylation in the activation segment [15]. In summary, these features indicate that d*m*Obsc-PK1 adopts an overall conformation similar to that of canonical kinases in the active state.

### 2.3. *Dm*Obsc-PK1 has a modified ATP-binding pocket that binds ATP with high affinity in the absence of cations

**Figure 3.**
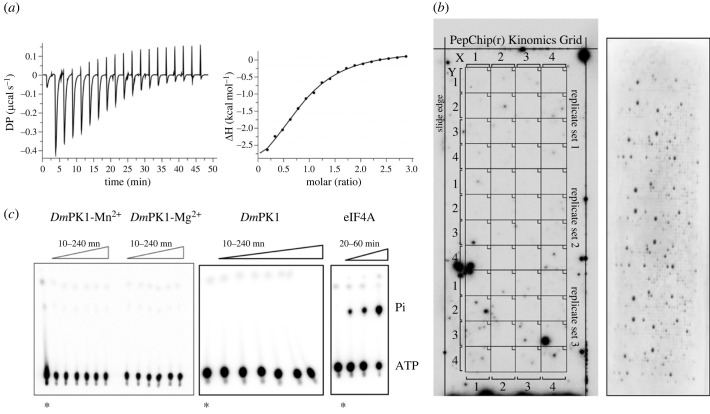
*dm*Obsc-PK1 binds, but does not process, ATP. (*a*) ITC measurement of ATP binding by *dm*Obsc-PK1 in the absence of cations. (The corresponding negative control is provided in electronic supplementary material, figure S4A). (*b*) Testing of phosphotransferase activity of *dm*Obsc-PK1 (left) on the PepChip kinomic array (Pepscan) using [λ-32P]-ATP (incubation conditions as described in methods). No phosphotransfer activity was detected in this assay (spots correspond to excess label remaining after washing). As expected, a Src kinase phosphotransfer assay (right) performed as positive control on the same array batch displayed positive signal from substrate peptides in triplicate. (The Src kinase assay used 1/10 sample concentrations during incubation respect to quantities employed for *dm*Obsc-PK1). (*c*) *dm*Obsc-PK1 does not display ATPase activity on [λ-32P]-ATP. The positive control eIF4A is included for comparison.

A structure-sequence analysis of *dm*Obsc-PK1 reveals that this is a pseudokinase with a degenerated ATP-binding pocket and active site (figure 2*e*). In its ATP-binding pocket, *dm*Obsc-PK1 displays canonical GxGxxG and AAK motifs (containing the essential lysine) and the DFG signature sequence (where the aspartate residue chelates the magnesium ion commonly required for catalysis). Also involved in cation coordination is a conserved asparagine residue in the catalytic loop motif [41]. This residue, however, is an aspartate in *dm*Obsc-PK1 (figure 2*e*). As this exchange has unknown functional consequences, we measured the binding of ATP to *dm*Obsc-PK1 using isothermal titration calorimetry (ITC) (figure 3*a*; electronic supplementary material, figure S4A). The measurements, which were performed in the absence of Mg^2+^ in triplicate, yielded a dissociation constant, *K*_d_, of 61.8 ± 4.2 µM. The value revealed that *dm*Obsc-PK1 binds ATP with high affinity in a metal-independent fashion. As the affinity for ATP is comparable to that of canonical active kinases [42] and considering the physiological concentration of ATP in cells (1–5 mM) [43], we concluded that *dm*Obsc-PK1 can bind ATP in muscle *in vivo*. Furthermore, the measurement of ATP complexation using the extended variant *dm*Obsc-PK1^ext^ was also indicative of strong binding (electronic supplementary material, figure S4B), which further demonstrated that in *dm*Obsc-PK1 the C-terminal sequence does not block ATP binding as observed in titin-like kinases.

To reveal the molecular basis of ATP complexation by *dm*Obsc-PK1, we elucidated its liganded crystal structure to 1.9 Å resolution using co-crystallization (table 1; figure 2*c*). Unexpectedly, electron density maps revealed ADP bound to the kinase (electronic supplementary material, figure S5), suggesting that ATP degradation had occurred over the lengthy crystallization period. Even though ITC had revealed that *dm*Obsc-PK1 binds ATP with high affinity in the absence of metal, crystallization was performed in the presence of Mg^2+^ ions. Nonetheless, no cation candidates could be identified in electron density maps, confirming a metal independent binding. In canonical kinases, magnesium coordinates the phosphate groups in ATP [41]. In *dm*Obsc-PK1 this function is performed instead by lysine K3310, whose side chain conformation is restrained and fixed in space by a salt bridge formation with aspartate D3311, the residue substituting the conserved asparagine in the catalytic loop (figure 2*c,e*). A weaker interaction between K3310 and aspartate D3326 from the DFG motif is also present. To analyze the conservation of the atypical aspartate and lysine residues in UNC-89/Obsc PK1, we performed a sequence analysis that explored over 1100 sequences of PK1 kinases from obscurin proteins (using BLAST; https://blast.ncbi.nlm.nih.gov). This revealed that *Drosophila* PK1 is a close representative (greater than 50% sequence identity) of PK1 kinases across arthropods, including wide lineages such as insects, spiders and aphids. In arthropods, residue D3311 is always a D/E residue, while lysine K3310 is either lysine or glycine (electronic supplementary material, figure S6). This reveals that atypical ATP binding is a conserved feature of PK1 in the arthropod phylum.

Other than having evolved a metal-independent coordination, ATP binding by *dm*Obsc-PK1 displays the regular features of canonical kinases [38,39,41]. Namely, the adenosine group of ATP completes the C-spine (figure 2*b*) and the *α* and β-phosphate groups are coordinated by the conserved lysine K3215 in strand-β3 (figure 2*c*). More globally, nucleotide binding does not induce either global or local conformational changes in *dm*Obsc-PK1, with apo and ADP-complexed crystal structures being equivalent (RMSD_C*α*_ = 0.18Å; figure 1*b*). Notably, conformational changes induced by nucleotide-binding are also not observed in other DMT kinases, including an absence of conformational variability in helix *α*C [15]. This suggests that *dm*Obsc-PK1 shares the pattern of DMT kinases, in which conformational transitions commonly associated with active/inactive states are not known to occur.

### 2.4. *Dm*Obsc-PK1 has a highly diversified catalytic site

Adding to the divergent mode of nucleotide binding, the catalytic motif of *dm*Obsc-PK1 is also strongly degenerated (figure 2*e*). Here, the conserved aspartate residue is replaced by a chemically inert glycine residue, G3306. The lack of a side chain in glycine results in the opening of a cavity on the surface of the kinase (figure 2*d*). At the deep end of that cavity, aspartate D3326 from the DFG motif is located as well as aspartate D3311 (that replaces the conserved asparagine of the catalytic loop, as described above). A BLAST sequence analysis showed that the glycine residue in the catalytic motif of *dm*Obsc-PK1 is strictly conserved across arthropods (electronic supplementary material, figure S6). The strong conservation of this cavity suggests that this kinase might accommodate in its active site amino acid side chains other than Ser/Thr or other metabolites. Thus, we explored the chemical entities that could bind into the cavity using in silico screening of chemical libraries; namely, EFI's Amino Acids and their derivatives (https://efi.igb.illinois.edu) and BMDB's Bovine Metabolome (https://efi.igb.illinois.edu) libraries, downloaded from ZINC15 (https://zinc15.docking.org) and docked to the kinase cavity with Autodock Vina 1.1.2 (https://vina.scripps.edu). This approach confirmed that the cavity is sufficiently large to accommodate metabolites as well as the side chain of large residues such as arginine and aromatic amino acids. Thus, it is tantalizing to envision that this conserved feature has evolved to accommodate unconventional binders. Unveiling whether such binders could act as atypical substrates, support the scaffolding role of the kinase or enable it to sense and respond to yet unidentified metabolic stimuli requires future investigation.

### 2.5. *Dm*Obsc-PK1 is a pseudokinase that lacks canonical kinase activity

While the glycine residue in the catalytic loop, G3306, cannot support phosphotransfer, we questioned whether the presence of aspartate D3311 at the bottom of the formed cavity might be a compensatory residue exchange that could preserve activity. To investigate this question and given that *dm*Obsc-PK1 substrates are not known, we replaced specificity with degeneracy by using a PepChip kinomic array (Pepscan), which contains 1024 eleven-mer peptides of known phosphorylation sites arranged in triplicate. We considered that this array contained sufficient sequence diversity and degeneracy to enable detecting phosphotransfer. Thus, we performed a kinase activity assay on this peptide array using radiolabelled *γ*32-ATP at 25°C. Because the degenerated active site of *dm*Obsc-PK1 suggested this kinase to be inactive or exhibit low levels of activity, the assay was performed in conditions of large excess. Namely, 1 µg of total recombinant purified kinase (10 fold over the recommended amount for an active kinase) was added to the array for a 6-hour incubation period (3 times the standard incubation time), with detection using a 96-hour exposure time. Results from the PepChip kinomic array did not reveal any positive hits for *dm*Obsc-PK1, with only irregular background signal being detected (figure 3*b*). In addition, we confirmed that the kinase does not undergo autophosphorylation by using intact mass determination mass spectrometry (electronic supplementary material, figure S7).

We asked next whether *dm*Obsc-PK1 is capable of intrinsic ATP processing by displaying ATPase activity. To test this, we incubated *dm*Obsc-PK1 with *γ*32-ATP in the presence of either Mg^2+^ or Mn^2+^ as well as in the absence of divalent ions. Thin layer chromatography was then used to separate the *γ*32-ATP substrate from *γ*32-P groups freed during putative ATP hydrolysis. The results demonstrated that no detectable release of the *γ*32-P product had taken place and, furthermore, that the initial *γ*32-ATP amounts were maintained indicating an absence of both ATPase and autophosphotransfer activity (figure 3*c*). As expected, the assay revealed significant *γ*32-ATP turnover by the eIF4A complex, which has well established ATPase activity [44] and was used here as a positive control. Taken together, our data indicate that *dm*Obsc-PK1 lacks canonical kinase activity.

### 2.6. The two kinase domains, PK1 and PK2, from *dm*Obsc interact

**Figure 4.**
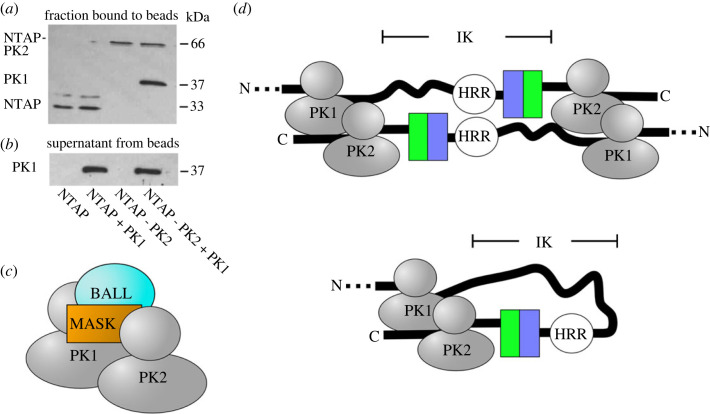
Interaction of PK1 and PK2 kinases from *dm*Obsc. Extracts of *Drosophila* thoraces containing muscles expressing NTAP or NTAP-*dm*Obsc-PK2 were added to IgG-agarose beads and the beads were then incubated with *dm*Obsc-PK1; (*a*) Western blot of the fraction bound to IgG beads. (*b*) Supernatant from IgG beads. *dm*Obsc-PK1 binds to NTAP-*dm*Obsc-PK2, but not to the control NTAP. (*c*) Schematic representation of molecular interactions at the *dm*Obsc PK1/PK2 locus. (*d*) Alternative models of possible modes of PK1/PK2 interaction in the sarcomere involving either intermolecular contacts across individual UNC-89/Obsc proteins (above) or intramolecular contacts within a same protein chain (below).

The apparent inactivity of *dm*Obsc-PK1 led us to consider that its function might align with that attributed to other inactive pseudokinases, which often act as protein-protein interaction scaffolds [45]. Specifically, pseudokinases often regulate canonical kinases, either promoting or attenuating their phosphotransfer activity. In this respect, the dual kinase tandem of *dm*Obsc resembles that occurring in the non-receptor Janus tyrosine kinase family (JAKs) [46]. JAKs have two nearly identical kinase domains that occur in tandem separated by a linker sequence of approximately 17 aa, where an inactive pseudokinase domain (JH2) is N-terminal to an active tyrosine kinase (JH1). Here, the pseudokinase domain negatively regulates the activity of the catalytic kinase. UNC-89/Obsc also contains two kinase domains in its chain where the PK1 pseudokinase is also located N-terminally to the predictably active PK2 kinase, but both domains are separated by a flexible 180 aa IK sequence and an Ig-FnIII tandem (figure 1*a*). Despite their distance in the polypeptide chain, we queried whether PK1 and PK2 in UNC-89/Obsc could act as a dual kinase system resembling the functional principle of JAKs, where PK1 and PK2 might be interacting partners. To test this interaction, *dm*Obsc-PK2 was expressed recombinantly in the muscles of flies *in vivo*. Extracts of the thorax, which is mainly composed of flight muscle, were incubated with recombinant *dm*Obsc-PK1. *dm*Obsc-PK2 in the muscle extract was immobilized on beads and shown to bind *dm*Obsc-PK1 (figure 4*a,b*). Interestingly, a recent report suggests that UNC-89 presents an overlapping, antiparallel arrangement in the M-line of *Drosophila* IFM, where the C-termini of two UNC-89/Obsc molecules overlap [47]. This points to the likelihood of an inter-chain kinase complex formation in muscle *in situ*, although the possibility of an intra-molecular interaction cannot be discarded (figure 4*d*). Unfortunately, efforts to either isolate or recombinantly produce *dm*Obsc-PK2 in our study were unsuccessful so that the extent of the regulation that PK1 might exert on PK2 could not be established here. An important implication of the direct interaction of PK1 and PK2 kinases, however, is that it brings its respective cellular binders in spatial proximity and, thereby, promotes the cross-talk of signalling pathways. *dm*Obsc-PK1 interacts with Ball kinase (Y2H assay) and MASK (TAP assay), while PK2 interacts with MASK (Y2H and TAP assay) [30]. Thereby, the PK1/PK2 interaction can be expected to position Ball kinase in the vicinity of PK2, possibly forging a functional link between these two kinases (figure 4*c*). In brief, our findings lead us to propose that the dual kinase system of obscurin proteins constitutes a functional unit, which likely results in the formation of a signalling node in its cellular context *in vivo*.

Finally, we aimed to gain an insight into the intrinsically disordered IK segment. Sequence-based bioinformatics prediction of secondary structure suggests the existence of a helix-rich (HRR) region spanning the C-terminal half of the IK sequence (figure 1*a*). Since a three-dimensional-fold could not be predicted for this region, we attempted to produce the HRR sample recombinantly for experimental analysis. However, a soluble protein product could not be obtained, supporting the view that the region does not adopt a stable three-dimensional-fold. Predictive three-dimensional-models for this region calculated with AlphaFold also did not display a compact fold, but formed an extended chain rich in helical secondary structure (electronic supplementary material, figure S9). Helices exhibit excellent elasticity, being able to undergo rapid mechanical unfolding-refolding transitions. In helices, the main chain hydrogen bonds align with the helical axis that, in turn, aligns with the force vector during stretch. As a result, helices undergo longitudinal shearing, behaving as constant force springs that unfold at moderate force [48,49]. Thereby, if confirmed, a HRR segment would contribute to the mechanical response of the IK region during muscle function. Taken together, the findings of our study lead us to suggest that PK1/PK2 form a pseudokinase/kinase dual system, where PK1 predictably acts as regulator of PK2 and where the connecting IK segment likely acts as an entropic spring, conceivably with a regulatory role on the kinase interaction. Thus, we propose that the PK1-IK-PK2 segment works as an integrated, functional unit in the sarcomere.

## 3. Discussion

Obscurins are sarcomere-associated, filamentous proteins that contain a distinct dual kinase region in C-terminal position. Our study of the obscurin homologue UNC-89/Obsc from *Drosophila* reveals that in this member of the family the N-terminal kinase, PK1, is an inactive pseudokinase that can interact with the C-terminal kinase, PK2. *dm*Obsc-PK1 presents general features typical of the DMT class of CAMK kinases; namely, it has a short helical C-terminal tail that folds against the kinase domain, constitutively formed spines, a pre-formed salt bridge between the conserved lysine in strand-β3 and the glutamate from helix *α*C, and it does not undergo conformational changes upon nucleotide binding. However, its ATP-binding pocket and catalytic site diverge notably from those of canonical protein kinases that perform phosphotransfer catalysis on peptidic substrates [41]. In the ATP pocket, the difference is localized to two residues in the catalytic loop; namely the conserved asparagine and its preceding residue, which in *dm*Obsc-PK1 are a KE motif. This alteration confers *dm*Obsc-PK1 the ability to bind ATP with high affinity in the absence of magnesium (figure 3*a*, electronic supplementary material, figure S4). According to a pseudokinase classification based on nucleotide/cation binding and processing, *dm*Obsc-PK1 is a class 2 pseudokinase [50]. ATP binding in the absence of a metal cation is also observed in other pseudokinases, such as CASK [51], TRB2 and ULK4 [52] and MLKL [53]. Interestingly, in TRB2 (and TRB pseudokinases in general) and ULK4 a lysine or arginine replaces the canonical asparagine of the catalytic loop and an arginine residue is observed in the preceding position where *dm*Obsc-PK1 displays a lysine (figure 2*e*). Magnesium regularly acts as an obligate cofactor for ATP-binding and phosphotransfer in protein kinases and its absence is usually linked to a loss in activity. However, some pseudokinases have been reported to perform phosphotransfer in the absence of a cation cofactor. For example, CASK, TrpB2 and TrpB3 have been shown to undergo autophosphorylation [51,52]. For *dm*Obsc-PK1, neither autophosphorylation, nor phosphotransfer or ATPase activity could be observed (figure 3, electronic supplementary material, figure S7), although the possibility of *dm*Obsc-PK1 having basal activity observable only after lengthy incubation times (such as those typically required for crystallization) cannot be discarded at present.

The active site of *dm*Obsc-PK1 also shows strong variations; namely, the catalytic base aspartate is replaced by a chemically inert glycine residue, where the lack of a side chain opens a sizeable cavity on the kinase surface (figure 2*d*). This change is accompanied by the exchange of the conserved asparagine in the catalytic loop for an aspartate residue, located at the bottom of the newly created cavity (figure 2*c,d*). Our data indicate that *dm*Obsc-PK1 does not show conventional phosphotransfer activity, in agreement with the fact that in kinases mutating the catalytic aspartate to alanine normally abolishes activity (e.g. [54]). Nonetheless, the strict conservation of the active site cavity (electronic supplementary material, figure S6) suggests that this is a functional feature. In this regard, pseudokinases with divergent folds and active sites have been found to be capable of performing atypical catalyses; namely glutamylation [55–57] and AMPylation [58]. In glutamylation, an isopeptide bond is formed between the amino group of free glutamate and the γ-carboxyl group of a glutamate residue in the substrate protein. In AMPylation, AMP is transferred from ATP to Ser, Thr or Tyr residues on a protein substrate. Whether the newly created cavity might support unexpected substrates or be exploited in a scaffolding role in this pseudokinase prompts future investigation.

Inactive pseudokinases often function as scaffolds for the assembly of protein complexes. In this respect, a well-established function of pseudokinases is their regulation of bona fide kinases [45]. Well characterized examples of this regulatory mechanism are pseudokinase/kinase pairs: JH2/JH1 Janus Kinases [46], HER3/EGFR, IRAK3/IRAK4 and KSR/RAF (recently reviewed in 45). It is possible that *dm*Obsc-PK1 might also act as an allosteric regulator of the C-terminal *dm*Obsc-PK2 and this view is supported by our finding of a direct interaction between the two kinases. A *dm*Obsc PK1/PK2 assembly would also aggregate Ball kinase and MASK (figure 4*c*). Future studies will be required to clarify the functional relations between constituents of this assembly in the sarcomere.

An interesting question relates to the stimulus sensed by *dm*Obsc-PK1 in the cell. Nucleotide binding does not induce a conformational transition in this pseudokinase, so that this event is unlikely to be of regulatory significance. An alternative hypothesis is that a conformational switch in *dm*Obsc-PK1 is induced mechanically by sarcomeric stretch, as attributed to titin and twitchin kinases [19,21–23]. However, TK and TwcK are the only known examples of CAMKs with developed mechanosensory flanking tails. Crystal structures of other members of the family - as e.g. CAMK-IIg, DAPK and CASK - revealed instead a short, C-terminal tail confined to helix *α*R1 and followed by a disordered sequence that often includes a calmodulin target motif (electronic supplementary material, figure S10). Also *dm*Obsc-PK1 displays a short C-terminal tail restricted to helix *α*R1, even though the possibility remains of further down-stream sequence segments within the IK region acting as kinase regulators by yet unidentified mechanisms. Regardless, the short helix *α*R1 element is of unlikely significance in stretch-response and lead us to conclude that *dm*Obsc-PK1 is not a bona fide force sensor in the myofibril. It is likely, however, that in UNC-89/Obsc force sensing is performed by the intrinsically unstructured IK sequence, where the *C. elegans* counterpart has been found to behave as an entropic spring [34]. In this regard, the predictive identification using bioinformatics methods in this study of a helix-rich region (HRR) within the C-terminal half of the IK sequence is of relevance to its mechanics. Furthermore, it is tantalizing to envision that mechanical signals might regulate the PK1-IK-PK2 node by means of the IK segment altering the interaction between PK1 and PK2 in a force-dependent manner.

**Figure 5.**
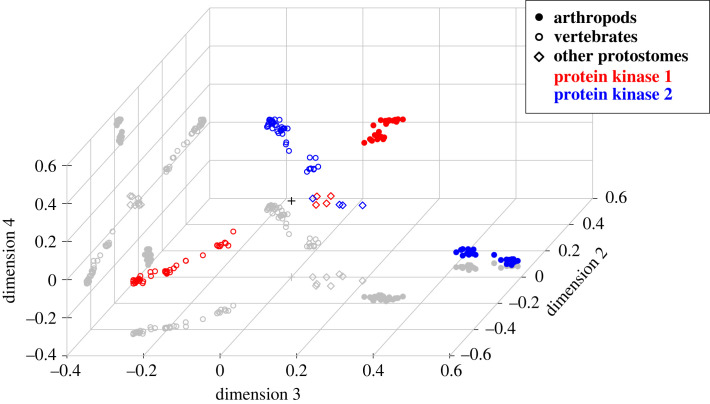
PaSiMap cluster analysis of PK1 and PK2 sequences from UNC89/Obsc and vertebrate Obsc proteins. An interactive version of this PaSiMap cluster map is provided as electronic supplementary material, where the identity of each data point can be queried.

Finally, to identify relations between *dm*Obsc-PK1 and other obscurin kinases, we performed a 3D-mapping of sequence similarities for 70 sequences of PK1 and PK2 domains from UNC89 and obscurin proteins from vertebrates, arthropods and nematodes using PaSiMap [59] (electronic supplementary material, table S1). PaSiMap represents protein sequences as coordinates in multidimensional space based on their pairwise similarities, where coordinates represent vectors from the origin. The angle of a vector reflects the systematic differences specific to a given sequence respect to others in the set under study, while the vector length from the origin reflects the strength of the signal, i.e. how well defined the systematic differences are. The closer to the origin a sequence is represented (i.e. the shorter its vector is), the less similar this sequence is to the ‘prototype of the group’ (longest vector with a similar angle). Strikingly, PaSiMap analysis revealed clearly segregated clusters corresponding to PK1 vertebrates, PK1 arthropods, PK2 vertebrates and PK2 arthropods. The clusters were equidistant in space, roughly along the mid-axes of a tetrahedral pyramide (figure 5; electronic supplementary material, data). This indicates that each kinase group is equally distinct and that the differences between PK1 kinases from different phyla is as large as between PK1 and PK2 kinases. In other words, kinases segregate with phyla and position within the tandem. Interestingly, PaSiMap maps show nematode sequences for both PK1 and PK2 located close to the origin, suggesting that these sequences are heterogeneous and present unique features in these organisms. Overall, the PaSiMap analysis points to a notable diversity in obscurin kinases and highlights the need of further molecular characterization of this kinase group.

## 4. Methods

### 4.1. Cloning

Variants of *dm*Obsc-PK1 (UniProtKB A8DYP0) spanning residues 3186–3480 (termed *dm*Obsc-PK1 in this study) and 3186–3495 (*dm*Obsc-PK1^ext^) were cloned into the vector pOPINF (Oxford Protein Production Factory, UK), which adds an N-terminal His_6_-tag and a PreScission protease 3C cleavage site prior to the insert. The variant spanning residues 3186–3480 was selected from a large construct screening (electronic supplementary material, figure S1) and corresponds to the crystal structure in this work. Clones were confirmed by sequencing. A codon-optimized version of the DNA coding for the predicted helix-rich region (*dm*Obsc-HRR) (UniProtKB A8DYP0; residues 3533–3652) was commercially synthesized (Geneart) and cloned into the vector pOPINM (OPPF, UK), which fuses a His_6_-tag, a maltose-binding protein and a PreScission protease 3C cleavage site N-terminally to the insert.

### 4.2. Protein production

Expression plasmids were transformed into *E. coli* Rosetta *pLacI(DE3)* (Novagen) and cultures grown in 2YT media supplemented with ampicillin (50 µg ml^−1^) and chloramphenicol (32 µg ml^−1^) up to an OD_600_ = 0.6 at 37°C. Protein expression was induced with 0.5 mM IPTG (isopropyl-β-D-thiogalactopyranoside) and cultures further grown for 16h at 18°C. Cells were harvested by centrifugation and lysed by homogenization in 20 mM Na_2_HPO_4_ pH 7.4, 500 mM NaCl, 20 mM imidazole, 3 mM β-Mercaptoethanol (*β*ME) containing an EDTA-free cocktail of protease inhibitors (Roche) and bovine deoxyribonuclease (Sigma). Lysates were clarified by centrifugation. Protein samples were purified using linear-gradient immobilized metal affinity chromatography (IMAC) in a 5 ml His-Trap HP column (GE Healthcare) and subsequently incubated with human rhinovirus 3C protease overnight. Samples were buffer exchanged into 20 mM Tris pH 8.0, 3 mM *β*ME and further purified by reverse metal affinity and Q-Fast Flow ion exchange chromatography (GE Healthcare). The resulting protein sample was subjected to full-mass determination analysis using mass spectrometry, which verified the absence of auto-phosphorylation. Prior to crystallization, *dm*Obsc-PK1 was buffer exchanged into 20 mM Tris pH 7.4, 50 mM NaCl, 3 mM *β*ME using either a Hi-Prep 26/10 or a PD-10 desalting column (GE Healthcare). Finally, the sample was concentrated to 13.5 mg ml^−1^ and stored at 4°C until further use.

The *dm*Obsc-HRR sample was expressed as described above. However, following cleavage of the His_6_-MBP tag with 3C-protease, the sample precipitated and became unviable, supporting the view that the HRR region of UNC-89 is unstructured.

### 4.3. X-ray crystallography

Crystals of apo *dm*Obsc-PK1 (residues 3186–3480) grew from solutions containing 1 M potassium sodium tartrate, 100 mM CHES pH 9.5, 200 mM lithium sulfate in Intelli-Plates (Art Robbins) using the sitting drop, vapour diffusion method at 18°C. Crystallization drops consisted of a 1 : 1 ratio protein:mother liquor and had a final volume of 400nL. Crystals typically appeared in 3 days. Crystals of *dm*Obsc-PK1 in complex with ATP were grown from media as those of the apo-form supplemented with 1mM MgCl_2_, 3mM ATP. Prior to cryo-crystallography, crystals were vitrified in mother liquor containing 20% [v/v] glycerol (supplemented with 2 mM MgCl_2_ and 4mM ATP for the ATP-complexed form).

X-ray diffraction data were collected at the DIAMOND synchrotron (Didcot, UK) on beamline I03 under cryo-conditions (100 K). Data processing used the XDS/XSCALE suite [60]. Phasing of the apo crystal form was by molecular replacement in PHASER [61] using the N- and C-terminal lobes of DAPK kinase (PDB code 3GU4) as independent templates. Initial atomic models were built using Arp/Warp [62] and further manual building was performed in COOT [63]. Model refinement was carried out in Phenix.refine [64] using isotropic B-factors and TLS refinement. The crystal structure of *dm*Obsc-PK1 complexed to ATP was solved using the structure of the apo form as template. Statistics for X-ray data processing and model refinement are given in table 1.

### 4.4. Prediction of three-dimensional-models using alphaFold

A three-dimensional-model of *dm*Obsc-PK1 encompassing the kinase flanking sequences (UniProtKB A8DYP0; residues 3159–3520) as well as a model of the HRR region were predicted using AlphaFold v. 2.2.0 [37]. AlphaFold calculations were executed locally at the University of Konstanz on a customized computer server (consisting of a processor AMD Ryzen Threadripper Pro 3975WX equipped with graphics card NVidia GeForce RTX3090 and 256GB RAM running the Ubuntu 20.04.4 operative system). The template database included Protein Data Bank entries up to 22-12-2021.

### 4.5. Isothermal titration calorimetry

Data were collected using MicroCal ITC 200 or Microcal PEAQ-ITC (Malvern) calorimeters at a protein concentration of 180 µM in 20 mM HEPES, 150 mM NaCl, 1mM TCEP, pH 7.5 at 298K. A solution of 2.7 mM ATP was added in 2 µl injections. Data fitting used a single site binding model in Origin Pro 7. The affinity of the interaction given by *K*_d_ = 1/*K*_a_ was calculated from triplicate measurements.

### 4.6. Phosphotransfer activity and ATP hydrolysis assays

*dm*Obsc-PK1 phosphotransfer activity was trialed using a PepChip Kinase Array (PepScan) incubated with 1 µg of PK1 protein in the presence of [λ-32P]-ATP in reaction buffer (50 mM HEPES pH 7.5, 150 mM NaCl, 3 mM DTT, 0.5 mM MnCl_2_, 0.5 mM MgCl_2_) at 25°C for 6h. Arrays were then exposed on a phosphorImager screen and results visualized on a Typhon 7500.

For ATP hydrolysis assays, *dm*Obsc-PK1 was incubated in reaction buffer (50 mM HEPES pH 7.5, 150 mM NaCl, 1 mM *β*ME, 0.5 mM MgCl_2_ or MnCl_2_) at 100 nM or 500 nM final concentration in the presence of [λ-32P]-ATP at 25°C. Reactions were quenched by the addition of equal volumes of HCOOH 1M. The reactions were then analysed by thin layer chromatography using PEI-cellulose (Whatman) and migration in 0.5 M LiCl, 1M AcOH. Following migration, plates were dried, exposed on the phosphorImager screen and results were visualized using a Typhon 7500.

### 4.7. Mass spectrometry

To measure intact mass, desalted protein sample was infused into the nano-electrospray source of a Waters Q-ToF micromass spectrometer at a flow rate of 50 µl h^−1^ via a gas tight syringe. The positive ion mass spectrum of the sample was scanned in the range *m/z* 80 to 2000 using a scan time of 1 s and a data acquisition time of 5 min. MassLynx MaxEnt1 was then used to convert the summed multiply charged spectrum to a molecular mass spectrum.

### 4.8. Pull-down assay from fly tissue

*dm*Obsc-PK2 was expressed in flies as previously described [30]. We used overexpression of PK2 in the native source organism as we could not achieve overexpression of soluble PK2 sample in other standard hosts (namely, *E. coli* and eukaryotic sf9 cells). For expression in flies, the coding sequence of *dm*Obsc-PK2 was cloned into the *pUAST-NTAP(GS)* vector [65] and transgenic flies were produced by microinjecting embryos. A fly line was also established expressing the control empty vector. Male flies were crossed with virgin females from the *Mef2-GAL4* line to produce expression in all muscles. *dm*Obsc-PK1 was produced recombinantly as described above.

Thoraces of 100 flies expressing NTAP-PK2, or control thoraces (NTAP), were homogenized and extracted with 0.3 ml buffer (0.15 M NaCl, 50 mM Tris pH 8.0, 1 mM EDTA, 0.1% Triton X-100) containing protease inhibitors for 30 min on ice. Samples were centrifuged at 16 000*g* for 30 min and dialysed against binding buffer (0.15 M NaCl, 20 mM MOPS, pH 7.0, 0.1% TritonX-100, 2 mM *β*ME) and centrifuged again. Extracts (6 µl, 50 µg) containing NTAP-PK2 or NTAP were incubated with 50 µl of IgG-agarose bead suspension (Sigma) in 300µL of binding buffer and rotated at 4°C for 2–3 h. Beads were washed with binding buffer three times and *dm*Obsc-PK1 (30 µg) was added to the beads, which were rotated at 4°C overnight. After washing, proteins were eluted from the beads with 100 µl SDS-PAGE sample buffer. Proteins bound to the beads and in the supernatants were run on 10% SDS gels; *dm*Obsc-PK1, NTAP-PK2 and the control NTAP were detected on western blots with anti-His antibody (diluted 1 : 10 000 Sigma). Blots were developed with chemi-luminescent substrate (Millipore).

### 4.9. PaSiMap sequence comparison

To collect protein sequences homologous to *dm*Obsc-PK1, a sequence search was performed with BLAST (https://blast.ncbi.nlm.nih.gov). This retrieved over 1100 sequences, of which 70 (listed in electronic supplementary material, table S1) were manually selected to represent mammals, fish, reptiles, amphibians, insects and nematodes. These were used as input for PaSiMap [59], which was executed through its web server (http://pasimap.biologie.uni-konstanz.de). To graphically visualize sequence similarities, a three-dimensional representation of the PaSiMap output was made in R (https://www.R-project.org). Similar to principal component analysis, in PaSiMap dimensions are ordered by the level in which they describe features of the dataset. The first dimension (1D) represents the most influential feature of the sequences, the second dimension (2D) maps a finer orthogonal feature, and so forth. In the analysis of PK1 and PK2 kinases, the variability in the sequence set was well accounted for by the first four dimensions, which were then used in visualization of the resulting groupings. Specifically, the sequences were mapped as coordinates in three-dimensional space using dimensions 2D, 3D and 4D, which reveal the finer detail of the clustering partition.

## Data Availability

Crystal structure coordinates and experimental structure factors have been deposited with the Protein Data Bank (entry 8AK2 and 8AK3 for the apo and ADP-complexed forms of *dm*Obsc-PK1, respectively). Diffraction images were deposited with Zenodo with access codes https://doi.org/10.5281/zenodo.6912575 [66] and https://doi.org/10.5281/zenodo.6912522 [67] for apo and ADP-complexed *dm*Obsc-PK1. The data are provided in electronic supplementary material [68].
